# Cerebral toxoplasmosis in a patient with myasthenia gravis and thymoma with immunodeficiency/Good’s syndrome: a case report

**DOI:** 10.1186/s12879-016-1801-y

**Published:** 2016-08-30

**Authors:** Sarah C. Sasson, Sarah Davies, Raymond Chan, Leo Davies, Roger Garsia

**Affiliations:** 1 Department of Clinical Immunology, Level 6 Laboratory Services Building, Royal Prince Alfred Hospital, Missenden Rd Camperdown, Sydney, NSW Australia; 2 Sydney Medical School, University of Sydney, Sydney, Australia; 3 Department of Infectious Diseases, Royal Prince Alfred Hospital, Camperdown, Australia; 4 Department of Neurology, Royal Prince Alfred Hospital, Sydney, Australia

**Keywords:** Myasthenia Gravis (MG), Toxoplasma, Thymoma, Immunodeficiency, Good’s Syndrome

## Abstract

**Background:**

Patients with thymoma with immunodeficiency (TWI)/Good’s syndrome characteristically have evidence of combined immunodeficiency including low or absent B-cells, hypogammaglobulinemia and defects in T-cell mediated immunity. These patients can present with common or opportunistic infections.

**Case presentation:**

A 54-year-old female was diagnosed with cerebral toxoplasmosis. This occurred on a background of metastatic thymoma previously treated with chemotherapy and myasthenia gravis (MG) treated with mycophenolate mofetil, monthly intravenous immunoglobulin (IVIG) and pyridostigmine. She reported recurrent herpes zoster infection. The patient had clinical and radiological progression of cerebral infection despite completing standard induction and maintenance therapy with sulfadiazine and pyrimethamine. Investigations found a complete absence of B-cells and evidence for hypogammaglobulinemia which, together with evidence of defects in T-cell mediated immunity and thymoma, lead to a diagnosis of TWI/Good’s Syndrome. The patient has undergone prolonged high-dose therapy for toxoplasmosis and a reduction in immunosuppression with no evidence of recurrent toxoplasmosis or flare of MG.

**Conclusions:**

TWI/Good’s Syndrome should be suspected in patients with thymoma and recurrent, persistent or unusual infections. If suspected serum immunoglobulins and lymphocyte subsets should be measured. These patients may need closer monitoring, higher dose and prolonged treatment of infections, and weaning of concurrent immunosuppression may be considered.

## Background

Thymoma, the most common tumour of the anterior mediastinum, is a rare malignancy of the thymic epithelium of unknown aetiology affecting males and females with approximately equal frequency. National Cancer Institute data from the USA suggests an incidence of 0.13/100 000 [1] and a peak in the 7th decade. Risk factors for the development of thymoma are largely unknown. Unlike other malignancies there is no evidence that common carcinogens such as tobacco and alcohol increase the risk of thymoma [1]. Similarly, no association has been shown between thymoma and other infections including human immunodeficiency virus (HIV) or Epstein-Barr virus infection [1]. There does appear to be an underlying genetic risk, with an increased incidence of thymoma in people of African-America, Asian and Pacific Island origin [1]. There is scant evidence suggesting thymoma occurs as a common second malignancy, including following treatment with ionizing radiation to the thorax [1].

Thymoma has been associated with a number of autoimmune conditions, with 30 % of patients developing an autoimmune condition by diagnosis or post-thymectomy [2]. It has been argued that thymoma-associated autoimmunity results from the T-cell precursor cells emigrating from a thymus expressing a dysregulated epithelium, with low expression of the autoimmune regulatory element (AIRE) [3] resulting in auto-reactive peripheral T-cells. A paucity of bone-marrow dendritic cells has also been described [3].

Thymoma has been most classically associated with MG where antibodies directed toward the acetyl choline receptor (AchR) result in post synaptic membrane destruction at the neuromuscular junction. Sixteen percent of patients with thymoma have a clinical diagnosis of MG, while an additional 22 % have AChR antibodies in the absence of clinical signs of disease [4] 15–20 % of patients with MG have thymic hyperplasia or thymomas. Interestingly, thymectomy does not provide absolute protection against developing MG and there have been reports of patients diagnosed with thymoma without MG or AChR antibodies, who have undergone thymectomy and have subsequently developed MG over 10 years later. It has been postulated this is due to the presence of auto-reactive T-cells already in the periphery. While MG is the most common thymoma-associated autoimmune disease other conditions include systemic lupus erythematousus, syndrome of inappropriate anti-diuretic hormone, acquired red-cell aplasia and bullous pemphigoid [2].

The association of thymoma with immunodeficiency has been less well appreciated. First described as Good’s Syndrome in 1955 [5] this condition was originally described as thymoma associated with low or absent B-cells, hypogammaglobulinaemia and defects in cell-mediated immunity. More recently this condition has been designated “thymoma with immunodeficiency” (TWI) and appears to affect males and females equally. Here we present the first report of a case of cerebral toxoplasmosis in a patient with MG and metastatic thymoma and clinical and laboratory findings consistent with TWI/Good’s Syndrome.

## Case report

The patient is a 54-year-old female who presented in September 2014 with headache, visual disturbance and right-sided facial weakness. There were no associated fevers or weight loss. Her past medical history included MG diagnosed in 1998 when she presented with ptosis and dysarthria. A thymoma was diagnosed and resected in 2003 but she subsequently developed pulmonary metastasis in 2011 and was treated with radiotherapy and chemotherapy including adriamycin, cisplatin and cyclophosphamide. Her past history also included hypertension, dyslipidaemia and a previous history of smoking. Of relevance, our patient contracted primary varicella zoster at the age of three months and had three episodes of herpes zoster (shingles) in her fifth decade. Medications on admission were: mycophenolate mofetil (MMF) 1 g PO BD, pyridostigmine 90 mg PO BD, prednisolone 12 mg PO OD, monthly intravenous immunoglobulin (IVIG) at a dose of 0.4 mg/kg for MG, atorvastatin 40 mg PO nocte, telmisartan 20 mg PO OD, multivitamin, calcium and cholecalciferol 1000 IU PO OD.

**Fig. 1:**
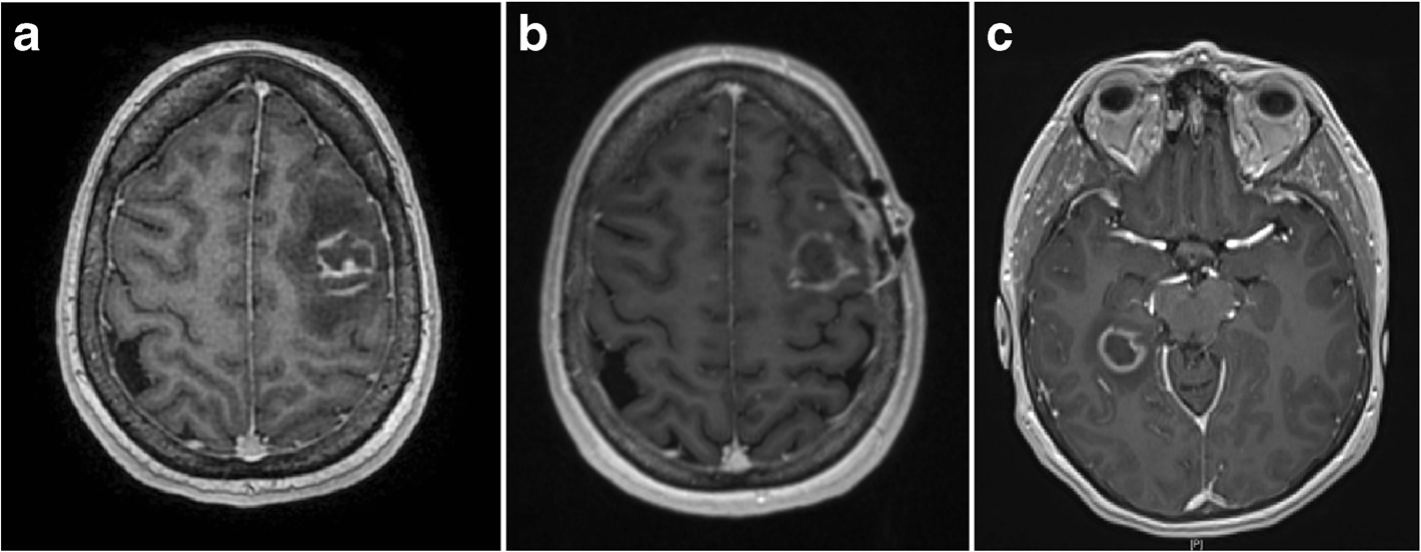
Progressive cerebral toxoplasmosis on interval neuroimaging. Serial magnetic resonance imaging (MRI) of the brain using sagittal T1 weighted images. At initial presentation there was (**a**) a solitary rim-enhancing lesion in the left posterior frontal lobe with moderate surrounding vasogenic odema and a 3.5 cm midline shift to the right. The patient had 4 weeks of therapy with oral sulfadiazine and pyrimethamine. Follow-up imaging showed (**b**) a persistent lesion in the left posterior frontal lobe as well as new miliary lesions in the cerebral hemispheres bilaterally and involvement of central grey structures including the right lentiform nucleus and thalamus. These findings were most consistent with disseminated toxoplasmosis. Four and a half months after the initial presentation and 4 weeks after a reduction to maintenance therapy the patient represented with nausea, headache and loss of consciousness and imaging showed (**c**) a new ring enhancing lesion in the right temporal lobe and persistent diffuse military nodules

Blood tests on admission are shown in Table 1. Of note there was a normal leukocyte count of 7.9 × 10^9^ with low lymphocytes 0.7 × 10^9^/L and monocytes 0.1 × 10^9^/L. A CT brain showed an irregular contrast enhancing lesion within the left frontal lobe with moderate associated vasogenic odema. A subsequent MRI confirmed the solitary solid/cystic rim-enhancing lesion in the left posterior frontal lobe with moderate vasogenic odema resulting in a 3.5 cm midline shift (Fig. 1a). In the setting of known metastatic thymoma this was thought to be most consistent with an intracranial metastasis. The patient underwent stereotactic left frontal craniotomy and excisional biopsy which showed toxoplasmosis on the basis of histopathology and polymerase chain reaction.

**Table 1:** Investigations at initial presentation and subsequent follow-up. Abnormal results are shown in bold. N/A = not available

Analyte	Value	Reference Range
Sodium	141	135–145 mmol/L
Potassium	4.4	3.5–5
Chloride	103	97–109
Bicarbonate	**23**	24–32
Urea	4.8	3–8 mmol/L
Creatinine	72	50-90micromol/L
Bilirubin	16	<21micromol/L
Albumin	**37**	38–48 g/L
Protein	**61**	62–80 g/L
ALP	43	30–130U/L
GGT	11	<35U/L
ALT	23	5–55U/L
AST	28	5–55U/L
WCC	7.9	4–10 × 10^9^/L
Hb	143	120–150 g/L
Platelets	300	150–400 × 10^9^/L
Neutrophils	7	2–7 × 10^9^/L
Lymphocytes	**0.7**	1–3 ×10^9^/L
Monocytes	**0.1**	0.2–1 ×10^9^/L
Eosinophils	0	0.0–0.1 ×10^9^/L
Basophils	0	N/A
Cryptococcal antigen	Negative	N/A
HIV-1/2 Ag/Ab test	Negative	N/A
IgG	11.1	6.39–15.6 g/L
IgA	0.73	0.7–3.12 g/L
IgM	**0.47**	0.5–3 g/L
IgD	<0.01	0.01–0.14 g/L
IgE	<2kU/L	0–120kU/L
CD3+ T-cell	**0.81 × 10^9**	1.2–2.7 × 10^9^
CD3 + T-cell (%)	**90 %**	58–85 %
CD19+ B-cell	**0**	4–19 %
CD19+ B-cell (%)	**0**	0.05–0.41 × 10^9^/L
CD4+ T-cell	**0.38 × 10^9**	0.4–1.32 × 10^9^/L
CD4+ T-cell (%)	42	31–58 %
CD8+ T-cell	0.41 × 10^9	0.22–0.74 × 10^9^/L
CD8 + T-cell (%)	**46 %**	11–37 %
CD16/56+ NK Cell	10 × 10^9	N/A
CD16/56+ NK Cell (%)	11	N/A

The patient commenced induction therapy for toxoplasmosis with sulfadiazine 1 g PO QID, pyrimethamine 75 mg PO OD and calcium folinate 15 mg OD according to local guidelines [6]. Her prednisone was increased to 25 mg PO OD and nystatin 1 ml PO QID was prescribed for oral candidiasis. She was discharged with a plan for 6 weeks induction course of anti-toxoplasma treatment and outpatient appointments with Neurology, Infectious Diseases and Neurosurgery. At review 3 weeks post-discharge, she complained of nausea, thought to be secondary to the sulfadiazine. Her medications were reduced to sulfadiazine 500 mg PO QID and pyrimethamine 50 mg PO OD.

Four weeks after initial presentation she underwent an MRI brain for post-neurosurgical follow-up. This revealed multiple new miliary lesions in the cerebral hemispheres bilaterally, which were predominantly cortical but with involvement of central grey structures including the right lentiform nucleus and thalamus (Fig. 1b). These lesions were not present on the initial MRI and were thought to represent disseminated toxoplasma. The patient was recommenced on sulfadiazine 1 g PO QID and pyrimethamine was maintained on 50 mg PO OD. Immunological review followed.

Further immunological investigations included flow cytometric evaluation of lymphocyte subsets which showed a CD4+ T-cell lymphopenia and complete absence of B-cells (see Table 1). Serum immunoglobulin levels showed normal IgG levels (consistent with replacement IVIG), low IgM and undetectable IgE and IgD. Although complicated by immunosuppression with MMF and treatment with IVIG, it was concluded that the lack of B-cells, serum immunoglobulins suggestive of an underlying hypogammaglobulinemia, and T-cell immunodeficiency as demonstrated by toxoplasma infection were all consistent with a diagnosis of TWI/Good’s Syndrome. The patient received a total of 12 weeks at induction level therapy for cerebral toxoplasmosis with sulfadiazine 1 g PO QID and pyrimethamine 50 mg PO OD. Following this the treatment was reduced to maintenance phase with sulfadiazine 1 g PO BD and pyrimethamine 25 mg OD.

**Fig. 2:**
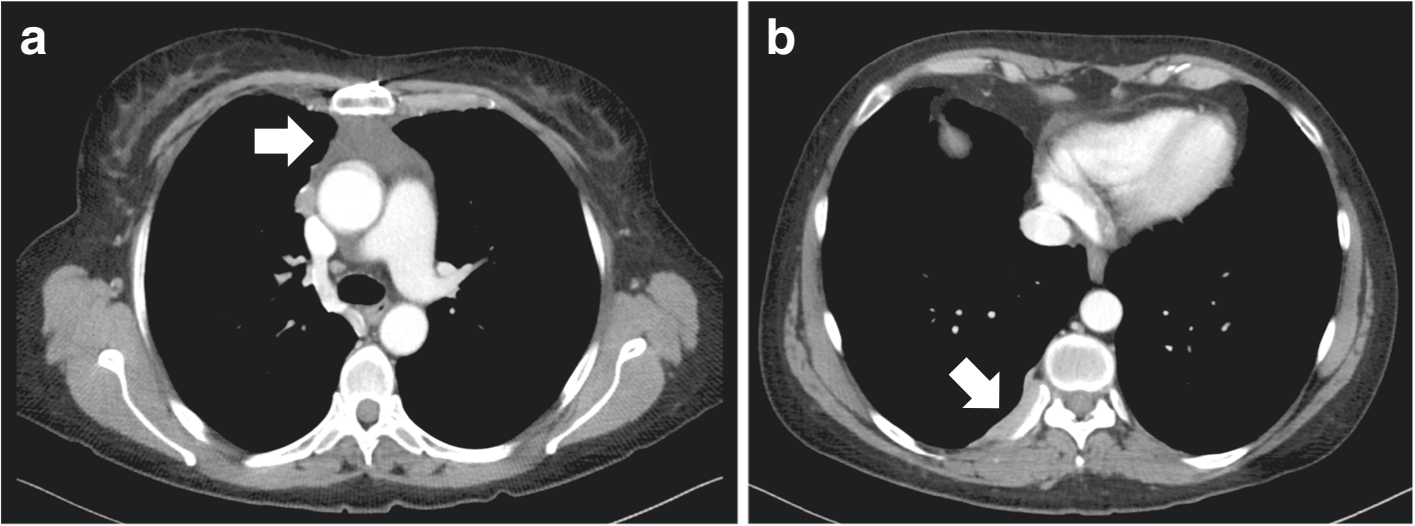
Metastatic thymoma on Computer Tomography (CT) of the chest. Evidence of metastatic thymoma at during follow-up as shown by (**a**) anterior mediastinal mass and (**b**) right sided pleural deposit

In February 2014, four and a half months after the initial presentation and 4 weeks after the second reduction in medications, the patient presented with acute onset nausea, headache and loss of consciousness. A CT of the brain showed a new ring-enhancing lesion in the right temporal lobe. An MRI of the brain confirmed the ring enhancing lesion in the right temporal lobe and the diffuse miliary nodules present in the previous MRI (Fig. 1c). These signs were considered to be consistent with persistent and progressive cerebral toxoplasmosis. She was recommenced on the induction dose of sulfadiazine 1 g PO QID and pyrimethamine 75 mg in addition to treatment intensification with atovaquone 750 mg PO BD. A cryptococcal antigen was negative as was HIV antigen/antibody screening. A CT of the chest showed stable anterior mediastinal and right sided pleural disease extending to the thoracic vertebrae exit foramen at the 9/10 level, with no evidence of new metastases (Fig. 2).

Two weeks following discharge a decision was made to withhold the MMF in order to promote cell-mediated immunity to the toxoplasma. Lymphocyte subsets repeated on multiple occasions consistently showed a complete absence of B-cells with fluctuating levels of CD4+ T-cells with a range 280–1100 × 10^9^/L. At the time of writing the patient remains stable on induction-dose sulfadiazine and pyrimethamine together with atovaquone therapy for toxoplasmosis, with MMF being withheld. The measureable cerebral lesions have reduced in size on MRI assessment and the miliary lesions have resolved. Her MG is stable and there have been no signs of progression of her metastatic thymoma. She is receiving localised radiotherapy to prevent nerve root and/or spinal cord compression at the thoracic 9/10 level, and continues to attend regular Neurology, Infectious Disease and Immunology outpatient clinics.

## Discussion

TWI/Good’s Syndrome is associated with low or absent B-cells, hypogammaglobulinaemia and defects in cell-mediated immunity but the mechanism of the immunodeficiency largely remains undetermined [7]. Indeed as late as 2005 this syndrome was considered a subset of the common variable immunodeficiency (CVID). It is postulated that the neoplastic thymic epithelium becomes disordered in TWI/Good’s Syndrome and results in a failure of thymic selection processes which may play a role in the associated T-cell deficiencies. We recently reported a case of recurrent thymoma where the precursor T-cell infiltrate was monoclonal [8], raising the possibility that T-cells generated in thymomas have a restricted T-cell receptor repertoire, that may contribute to immunodeficiency. How TWI/Good’s Syndrome results in hypoproduction of B-cells and hypogammaglobulinemia is not well understood. It is postulated that this may be the result of autoimmune T-cell destruction of B-cells or aberrant regulatory T-cell function. Similar to MG, immunological abnormalities in TWI/Good’s Syndrome are not corrected by thymectomy.

TWI/Good’s Syndrome most commonly presents between the ages of 40-70years and 47 % of reported world-wide cases have been reported from Europe. In the largest systematic review in this area, Kelesidis and Yang reported on the clinical laboratory and immunologic findings of 152 cases of TWI/Good’s Syndrome [7]. Patients presented with bacterial, viral, fungal and protozoan infections. The most common bacterial infections were recurrent sinopulmonary and other lung infections, bacteraemia and bacterial diarrhoea. The most common viral infections were herpes simplex virus and human herpes virus (HHV)-8. Candida infections were common and nine patients had the opportunistic pneumocystis jirovecii pneumonia (PJP). Of the parasitic infections giardia was the most common. There was one case-report of babesia microtii and one case of ocular toxoplasma [9].

In the same series of patients with TWI/Good’s Syndrome, all patients had an autoimmune condition with pure red cell aplasia being the most common (34.8 %) followed by MG (15.7 %) and oral lichen planus (12.4 %) [7]. In regards to abnormal laboratory findings all patients had hypogammaglobulinemia with the majority also displaying low or absent B-cells (87 %), anaemia (85 %) or CD4 + T-cell lymphopenia <360cells/μL (73 %) [7].

This case demonstrates the importance of considering immunological investigations in patients with thymoma who present with unusual infections, the difficulty in making a diagnosis of TWI/Good’s Syndrome and the complexity of clinical decision making in such patients. Our patient was a typical age for a patient with TWI/Good’s Syndrome. Her thymoma is notable for the fact that it had metastasised and there is no published data on whether the incidence of TWI/ Good’s Syndrome is higher in patients with metastatic thymoma.

In terms of making the diagnosis of TWI/Good’s Syndrome the pivotal finding in this case was a complete absence of B-cells. While our patient additionally had evidence of hypogammaglobulinemia, this finding was difficult to interpret due to monthly IVIG for MG. Additionally, the diagnosis of both cerebral toxoplasmosis and the three episodes of shingles in the previous 4 years provide evidence of impaired cell-mediated immunity. While it could be argued that the hypogammaglobulineamia and defects in cellular immunity could be related to the past history of metastatic thymoma, previous treatment with systemic chemotherapy and ongoing MMF, this does not account for the consistent finding of a complete absence of B-cells in this case which is striking and helps to confirm the diagnosis of TWI/Good’s Syndrome. The only other feasible explanation would be previous treatment with B-cell depletion therapy e.g. Rituximab which the patient had not received.

This case also illustrates the difficulty in clinical decision making regarding such patients. Our patient demonstrated progressive cerebral toxoplasmosis even when a standard 12 week induction therapy schedule was completed. While there is no international accepted gold-standard of treatment for cerebral toxoplasmosis, the combination of sulfadiazine and pyrimethamine has been shown to be as effective as other commonly used regimens such as pyrimethamine and clarithromycin, or trimethoprim-sulfamethoxazole with a cure rate of approximately 50 % [10]. The decision to add atovaquone as an additional antimicrobial had to be weighed against risks of patient tolerability and drug toxicity. The more difficult decision was that to withhold the MMF immunosuppression to aid cell-mediated immunity, a decision that risked relapse of MG which fortunately has not occurred. The patient remains on long-term high dose anti-toxoplasma treatment. There is little data to guide decisions on duration of treatment and ongoing decisions will have to be made on balance by the treating teams involved.

## Conclusions

To our knowledge this is the first report of cerebral toxoplasmosis in a patient with TWI/Good’s Syndrome. This case along with other work demonstrates the importance of undertaking immunological investigations in patients who have thymoma and infections. This should include serum immunoglobulins, lymphocyte subsets and IgG antibodies to toxoplasma and CMV in patients with confirmed TWI/Good’s Syndrome. Patients with hypogammaglobulinaemia should receive replacement IVIG and those with CD4 T-cells counts <200cells/μL should receive PJP prophylaxis. Additionally, live vaccines pose a significant threat to patients with TWI/Good’s Syndrome and should be avoided where possible. Unfortunately the prognosis of patients with TWI/Good’s Syndrome appears to be worse than other immunodeficiencies with one single centre study finding the 10-year survival for patients with CVID being 93 % but only 33 % in patients with TWI/Good’s Syndrome [11]. It is hoped that further recognition and aggressive management of patients with TWI/Good’s Syndrome along with a growing understanding of this condition will improve the overall survival.

## Abbreviations

AChR: Acetyl choline receptor
AIRE: Autoimmune regulatory element
BD: Twice daily
CD: Cluster of differentiation
CT: Computer tomography
CVID: Common variable immunodeficiency
HIV: Human immunodeficiency virus
Ig: Immunoglobulin
IVIG: Intravenous immunoglobulin
MG: Myasthenia gravis
MMF: Mycophenolate mofetil
MRI: Magnetic resonance imaging
OD: Once daily
PJP: Pneumocystis jirovecii pneumonia
PO: Orally
QID: Four times a day
TWI: Thymoma with immunodeficiency
USA: United States of America
